# RECESSION EVENTS AND SLEEP PROBLEMS IN MIDLIFE AND AGING ADULTS

**DOI:** 10.1093/geroni/igac059.1049

**Published:** 2022-12-20

**Authors:** Aarti Bhat, Jose Diaz, David Almeida

**Affiliations:** The Pennsylvania State University, State College, Pennsylvania, United States; The Pennsylvania State University, University Park, Pennsylvania, United States; The Pennsylvania State University, University Park, Pennsylvania, United States

## Abstract

Adverse economic events can negatively impact aspects of health, including sleep quality. Poor sleep can increase risk of developing or exacerbating health conditions such as cardiovascular and metabolic disease, cancer, and suicidal ideation. It is critical to examine how economic hardships may amplify health disparities in midlife and aging, a rapidly growing demographic in the U.S. This study examines the effect of recession hardships on sleep issues in midlife and aging adults using waves 2 and 3 of the Midlife in the United States study (MIDUS; N = 2602; M age = 63.47; 56.99% women; 15.76% Black). Participants reported chronic sleep problems experienced in the past year, alongside frequency of experiencing sleep disruptions (trouble with onset, maintenance, feeling unrested). Participants also reported economic impacts (financial, housing, and job-related) experienced in the aftermath of the recession; with 75.56% reporting at least one adverse recession event. Regression indicated that recession events were significantly associated with higher odds of chronic sleep problems and high frequency sleep disruptions in wave 3, even when controlling for sleep problems in wave 2. When examining race and age as moderators, Black participants who experienced adverse events were more vulnerable to chronic sleep problems than white participants, whereas age showed no significant interactions. Results indicate that adverse economic events can impact sleep quality for midlife and aging adults, and that policy mitigating economic effects on sleep may especially benefit Black adults. Subsequent analyses will examine the effect of recession events on daily sleep patterns.

